# An "Anti-Vivisection" Hospital

**Published:** 1897-06-12

**Authors:** 


### The Hospital

A Journal of

The Medical Sciences and Hospital Administration.

Vol. XXII.—No. 559.] [June 12, 1897.

### An "Anti-Vivisection" Hospital.

It has been said, apparently with absolute truth, that the boasted advancement of science of the Victorian era, and its constantly extending applications to the safety, the comfort, and the convenience of the public, are matters which depend for their permanence and for their continuance upon the lives of a very small number of persons. It is calculated that the simultaneous extinction of about two hundred physicists would be sufficient to bring back upon Europe a condition of mediæval barbarism, to rekindle the fires of religious persecution, and to arrest, probably for centuries, the progress of discovery. A merely literary education, even of a high order, renders no assistance in the elucidation of the mysteries of Nature; and the "commercial" education of the present day would be even still less useful with regard to them. Our Universities might furnish us with scholars comparable to Scaliger or to Erasmus, and with geometricians comparable to Archimedes or Apollonius; but neither class would be capable of recovering the lost secrets of modern work in the domain of ether, or of restoring physical knowledge to the position which it now occupies in the daily life of mankind. The united business aptitudes of the whole of the inhabitants of the City of London would be unable to arrive at the discoveries made by a single Wheatstone or by a single Faraday. The light of science does indeed shine upon the multitude, but the torches by which it is furnished are held aloft by the hands of a few.

We have been led to the foregoing observations by the sight of a small handbill, printed in bad type upon inferior paper, and proposing, as one of the methods of celebrating the Queen's "Diamond Jubilee," the establishment of a "National Anti-Vivisection Hospital," in which, "by the trust deed it shall be impossible to carry on vivisection in the schools, and for any vivisector to be on the staff." The "trustees" already appointed are said to be Lord Coleridge and two other persons unknown to fame, one of whom is a member of the medical profession, but is not at present on the staff of any hospital. He would, no doubt, be eligible for office in the new one.

Were it not for being aware how completely the whole case of the so-called "anti-vivisectionists" has been born and nourished in and upon falsehood, we should be puzzled to explain the pertinacity with which it is stated that "hospitals" are the chief centres of experimentation upon living animals. Such experiments are performed in physiological laboratories, and it is a matter of accident whether a physiological laboratory be connected with a hospital, in the sense of being contained within its buildings, or wholly independent of it. Examples of both kinds are well known; but, even when the building is attached to a hospital, it is rare for the active members of the staff, the physicians and surgeons engaged in the treatment of disease within the wards, to be themselves experimenters. The members of the former class thankfully avail themselves of all the knowledge which experimentation can furnish, but as mere questions of other demands upon their time, and of the advantages arising from division of labour, they seldom work in laboratories themselves. They are more apt to place before a colleague engaged in research their perplexities with regard to some problem encountered in the course of practice, and to seek from him the information necessary for its solution. The actual experimenter is usually a person whose active life is largely devoted to the investigation of vital phenomena, whether these be healthy or morbid; and whose trained skill enables him to take the most direct road to the information which he desires to obtain. It would be a matter of absolute indifference to him whether he worked in a room which formed part of hospital buildings, or in one which was wholly unconnected with them. He never wastes life, and practice enables him to attain his ends with a minimum of suffering to the subjects of his experiments. The word "vivisector," as applied to him, is a question-begging absurdity, because it covers only a small portion of his procedure, and because it would include many other procedures, some of which are not scientific in any sense. "Vivisection" only means, of course, an operation with a cutting instrument performed upon a living animal; and it not only includes nearly all surgical operations of a curative character, but also the bleeding of calves or of turkeys by the butcher or the farmer. It leaves untouched whole fields of physiological experiment, thermal, electrical, and other, for which cutting instruments are not required.

Assuming, what is a very large assumption, that the proposal for the new hospital is made seriously, and not as a mere attempt on the part of knaves to wheedle money out of fools, it appears to us that those who became responsible for its management would incur imminent risk of being convicted of manslaughter, if not of wilful murder. Everything which is worth knowing in modern medicine and surgery, everything which distinguishes them from the medicine and surgery of the time of Molière and of the century or two preceding him, is a direct result of experiments on living animals. If the managers of the new hospital are to be consistent, all this knowledge must be repudiated. Arterial bleeding must be dealt with by hot iron. Aneurisms must be suffered to enlarge and to burst without restraint. Compound fractures and operation wounds must no longer be treated antiseptically. The increase of mortality, alike in patients who were treated by fourteenth century methods, and in those who were not treated at all, would be likely to give rise to curious inquiry on the part of coroners ; and, even if the hospital managers permitted the employment of knowledge which had been acquired prior to the opening of their institution, they would soon be placed in difficult positions by neglecting to avail themselves of that which was acquired afterwards. Suppose, what is quite possible, that a method of curing cancer were to be discovered and established by experiment, and that the patients in the new hospital were systematically suffered to die for want of the application of the remedy.

